# Supplementary material for: singlecellVR: Interactive Visualization of Single-Cell Data in Virtual Reality
Source: Front Genet. 2021 Oct 28;12:764170. doi: 10.3389/fgene.2021.764170 (PMC8582280; doi:10.3389/fgene.2021.764170)

Supplementary Figure 2

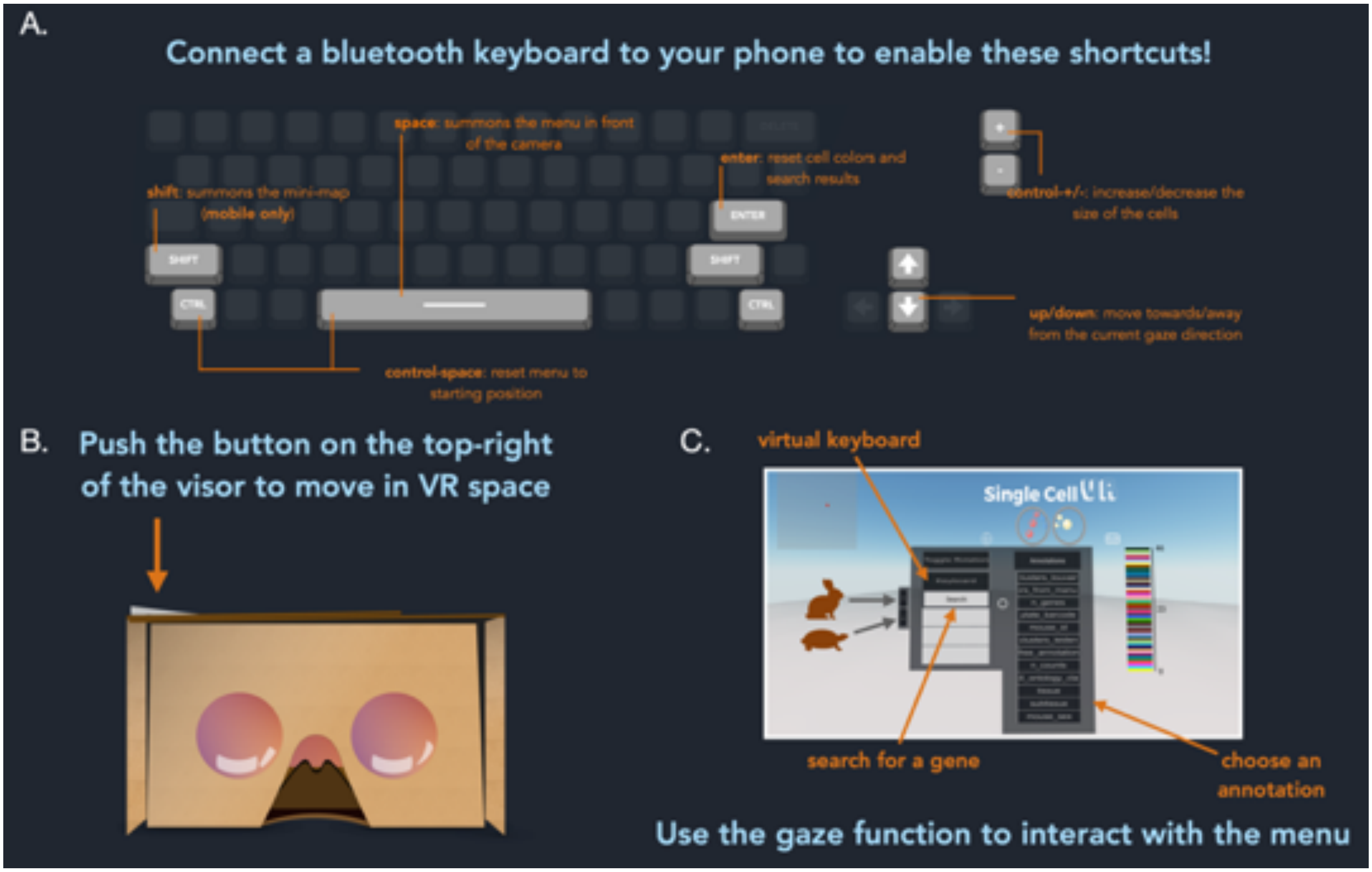

Supplement: Supplementary file 2 [file Image2.pdf]
