# Supplementary material for: singlecellVR: Interactive Visualization of Single-Cell Data in Virtual Reality
Source: Front Genet. 2021 Oct 28;12:764170. doi: 10.3389/fgene.2021.764170 (PMC8582280; doi:10.3389/fgene.2021.764170)
Supplement: Supplementary file 6 [file Image1.pdf]

Supplementary Figure 1

Step 1: fork the singlecellVR repository

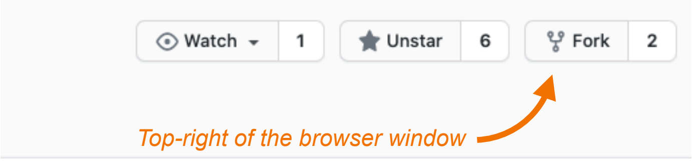

Step 2: clone your forked version and add any files

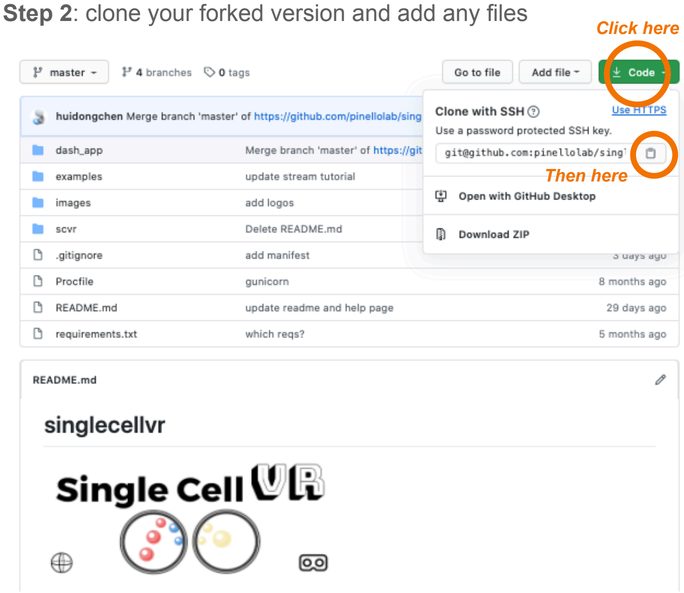

Step 3: create a pull request

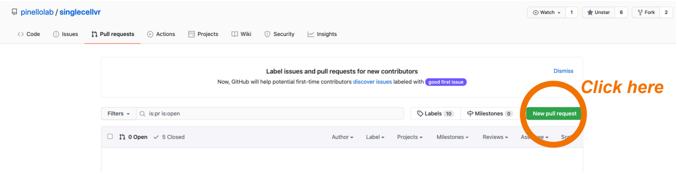

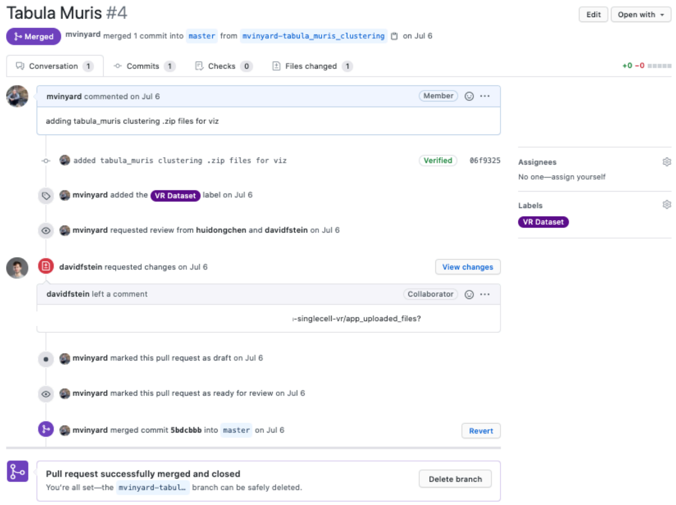

Step 4: submit and once approved, it will be added to the website for all!
